# Oral Eplerenone Versus Observation in the Management of Acute Central Serous Chorioretinopathy: A Prospective, Randomized Comparative Study

**DOI:** 10.3390/ph13080170

**Published:** 2020-07-29

**Authors:** Ramesh Venkatesh, Arpitha Pereira, Chaitra Jayadev, Vishma Prabhu, Aditya Aseem, Kushagra Jain, Bharathi Bavaharan, Naresh Kumar Yadav, Jay Chhablani

**Affiliations:** 1 Narayana Nethralaya, Department of Retina & Vitreous, 121/C, Chord Road, 1st ‘R’ Block, Rajaji Nagar, Bengaluru 560010, India; vramesh80@yahoo.com (R.V.); arpitha1988@gmail.com (A.P.); drchaitra@hotmail.com (C.J.); dr.vishmaprabhu@gmail.com (V.P.); aditya.aseem@gmail.com (A.A.); drkushagrajain@gmail.com (K.J.); bharathibavaharan@gmail.com (B.B.); vasudha.naresh@gmail.com (N.K.Y.); 2 Department of Medical Retina and Vitreoretinal Surgery, University of Pittsburgh School of Medicine, 203 Lothrop Street, Suite 800, Pittsburg, PA 15213, USA

**Keywords:** acute, central serous chorioretinopathy, eplerenone, contralateral eye

## Abstract

In this prospective, interventional case-control study, 58 patients with unilateral acute central serous chorioretinopathy (CSCR) were recruited. Patients ≥ 18 years age, presenting with first episodes of acute CSCR, were included. Acute CSCR was defined by the presence of subretinal fluid (SRF) and symptoms for <12 weeks duration with no clinical or imaging features of chronicity. Patients were alternately divided into treatment (Table Eplerenone 50 mg/day for minimum 1 month) and observation groups. Vision, SRF height and subfoveal choroidal thickness (SFCT) were checked at 1-, 2- and 3-months in both eyes of each group. Each group had 29 eyes. Mean age was 40.4 ± 7.1 and 43.3 ± 8.34 years in treatment and observation group, respectively. Mean symptom duration was 6.46 ± 1.45 and 5.87 ± 2.09 weeks, respectively. Vision improvement to 6/6 was seen in 92%, 100% and 100% cases in treatment group and 74%, 86% and 100% in control group at each visit, respectively. Complete SRF resolution in the treatment group was noted in 45%, 55% and 62% cases at each respective monthly visit. In the observation group, complete SRF resolution was noted in 10%, 21% and 31% at 1-, 2- and 3-month visits, respectively. SRF (*p* < 0.001) and SFCT (*p* < 0.001) reduction was noted in the affected eye of both groups. SFCT was reduced in the fellow eye after treatment (*p* = 0.005) compared to the observation group (*p* = 0.276). In conclusion, oral eplerenone achieves faster SRF resolution and vision improvement in acute CSCR. Additionally, it shows beneficial effects on the fellow eye.

## 1. Introduction

Central serous chorioretinopathy (CSCR) is characterized by serous detachment of the retina and/or retinal pigment epithelium (RPE) usually confined to the macula [1]. Men are more commonly affected, and the age of onset can range from the 4th–6th decade of life [1,2]. The common symptoms in acute CSCR include central vision loss, dyschromatopsia, central or paracentral scotoma, metamorphopsia and/or micropsia. The duration of subretinal fluid (SRF) accumulation lasts for <3 months in acute CSCR. It is a relatively benign condition with spontaneous absorption of SRF expected to occur in 2–3 months due to the healthy RPE [3]. Over one-third to one-half of the patients experience recurrent disease over the long term follow-up [1,4]. Persistent SRF may progress to chronic CSCR, also known as diffuse retinal pigment epitheliopathy, and lead to progressive and irreversible damage to photoreceptors, which may have a permanent effect on vision [1,5]. Thus, treatment modalities that could hasten the SRF absorption and prevent recurrences early in the disease to prevent vision loss are necessary. 

The pathogenesis of CSCR is multifactorial, extremely complex and poorly understood. Recent studies have suggested CSCR to occur as a result of choroidal vascular dysregulation. This is confirmed by the choroidal hyper permeability changes shown by fluorescein angiography and/or indocyanine green angiography [6,7]. Additionally, increased choroidal thickness has been demonstrated in both the affected and contralateral eyes when measured with optical coherence tomography (OCT) [8,9]. Newer studies have implicated the role of glucocorticoid and mineralocorticoid dysfunction in the pathogenesis of CSCR [10,11,12,13]. Increased choroidal vasodilatation and leakage have been noted following the activation of choroidal mineralocorticoid receptors in rat animal models; this phenomenon was inhibited by the use of mineralocorticoid receptor antagonists [14]. Spironolactone and eplerenone are both oral specific mineralocorticoid receptor antagonists approved for several systemic disorders and are commonly used for treating hypertension and congestive heart failure [15]. However, spironolactone, although effective, is associated with progestational and antiandrogenic side effects such as gynecomastia, impotence and abnormal menstrual cycles, which can limit its use. Eplerenone has a lower affinity for steroid receptors (e.g., progesterone and androgen), leading to higher tolerability and specificity [16,17]. 

The efficacy of spironolactone and eplerenone in the treatment of CSCR has already been well established. However, many of these studies have been done for the treatment of chronic CSCR [18,19]. A recently published multicentric clinical trial (VICI trial) from the United Kingdom studied the effects of oral eplerenone in patients with treatment-naïve CSCR of >4-month duration. It suggested that eplerenone was not superior to placebo treatment for improving visual acuity in CSCR. However, the paper did not study the effects of eplerenone on eyes with treatment-naïve acute CSCR cases <4 months duration [20]. There exists a paucity in the literature on the role of eplerenone and spironolactone in acute CSCR. In addition, studies have found that systemic mineralocorticoid receptor antagonists hasten SRF resolution in acute CSCR [21,22]. However, the effects of the drug on the contralateral eye have not been evaluated. 

Treatment is usually not required for acute CSCR cases; however, considering the young and working age group of the patients, a faster recovery and resolution of SRF using a safe rather than a disruptive approach with focal laser or photodynamic therapy would be preferable. 

Thus, in this prospective case-control study, we evaluated the efficacy of oral eplerenone compared to observation in eyes with acute CSCR. We also studied the effects of the drug on the choroidal thickness of both eyes. 

## 2. Results

In this prospective study, a total of 63 eyes of 63 patients met the inclusion criteria. However, five patients (three patients in the observation group and two patients in the treatment group) did not even complete their one-month follow-up visits and were dropped out from the study and the final analysis. Thus, the data of 58 eyes of 58 patients were included for analysis, and 29 eyes of 29 patients each were included in the treatment and observational group. The mean age and gender distribution between the two groups were similar. Mean duration of symptoms were 6.46 ± 1.45 weeks and 5.87 ± 2.09 weeks, respectively. Eyes in both groups had a presenting visual acuity of ≥6/36 except for one eye in the treatment group. As seen in Table 1, no significant differences in SRF height and SFCT were noted at baseline between the two groups.

### 2.1. Visual Acuity Changes during the Follow-Up Visits

During the monthly follow-up visits, visual acuity improvement to 6/6 was seen in 92%, 100% and 100% of cases in the treatment group at the 1st, 2nd and 3rd month visits, respectively. In the control group, visual acuity improvement to 6/6 was noted in 74%, 86% and 100% cases at the monthly three follow-up visits, respectively. All the eyes showed visual acuity improvement to 6/6 by two months in the treatment group and by three months in the observation group. 

### 2.2. Efficacy of Eplerenone on the SRF Height in the Study Eye

During the monthly follow-up visits, complete SRF resolution was seen on spectral domain OCT in 45%, 55% and 62% of cases in the treatment group at the 1st, 2nd and 3rd month visits, respectively. In the control group, complete SRF resolution was noted in 10%, 21% and 31% cases at the monthly three follow-up visits, respectively. The p values between the two groups at the 1st, 2nd and 3rd month visits were 0.007, 0.014 and 0.034, respectively (Table 1). Significant reduction in the SRF height was noted in both treatment (*p* < 0.001) and control groups (*p* < 0.001) (Table 2). 

### 2.3. Efficacy of Eplerenone on SRF Height and SFCT on Study Eye

**Figure 1:**
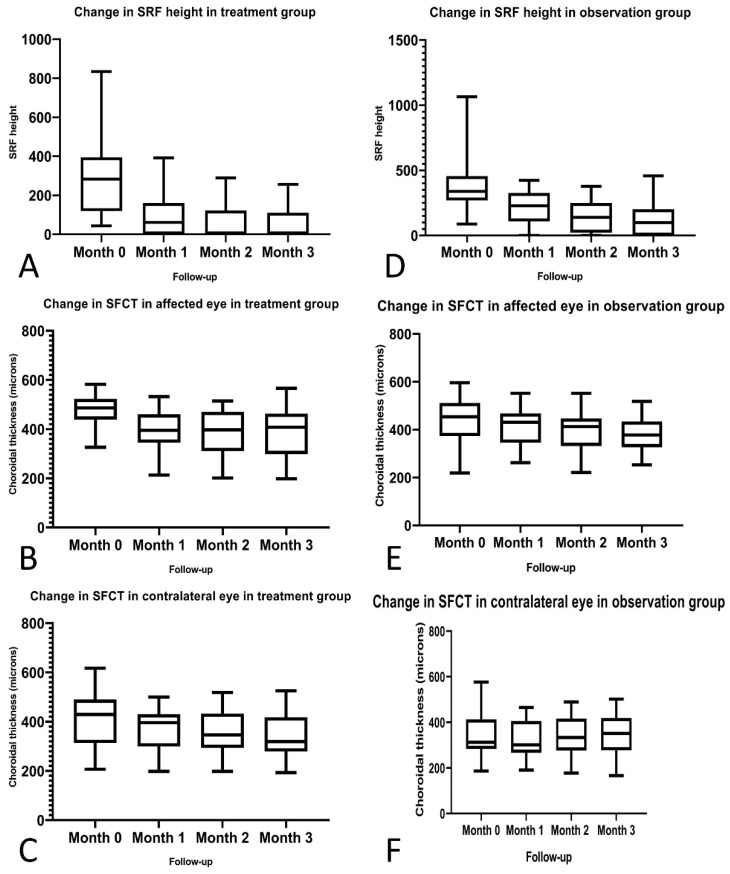
Changes in subretinal fluid (SRF) height and sub foveal choroidal thickness (SFCT) in both treatment and observation groups. (**A**–**C**): Box and whisker plots showing the change in the SRF height and SFCT in the treatment group at baseline, 1-,2- and 3-month visits. (**D**–**F**): Box and whisker plots showing the change in the SRF height and SFCT in the observation group at baseline, 1-,2- and 3-month visits.

**Figure 2:**
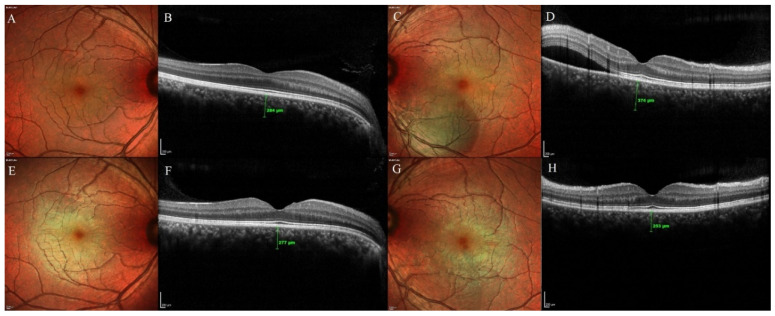
Changes in the subretinal fluid (SRF) height and sub foveal choroidal thickness (SFCT) of both eyes on optical coherence tomography in the observation group. (**A**,**B**): Multicolor and OCT image of the fellow eye at presentation. The SFCT at presentation was 284 µm. (**C**,**D**): Multicolor and OCT image of the study eye at presentation showing the SRF. The SFCT at presentation was 374 µm. (**E**,**F**): Multicolor and OCT image of the fellow eye at the 3-months final follow-up visit. The SFCT was 277 µm. (**G**,**H**): Multicolor and OCT image of the study eye at 3-months final visit showing the complete resolution of the SRF. The SFCT was 253 µm.

**Figure 3:**
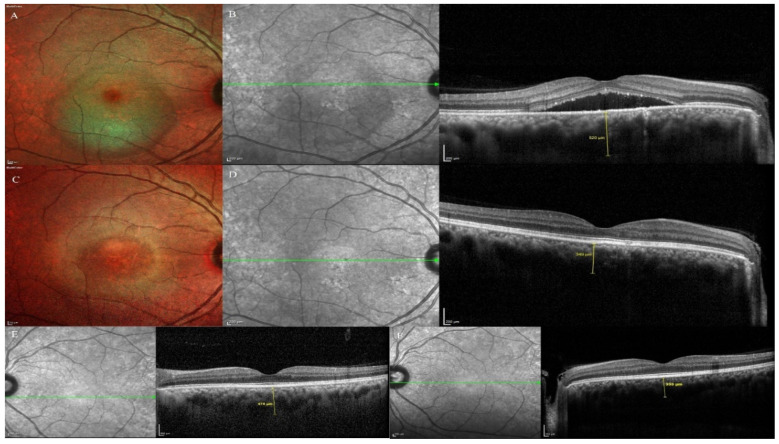
Changes in the subretinal fluid (SRF) height and sub foveal choroidal thickness (SFCT) of both eyes on optical coherence tomography in the treatment group. (**A**,**B**): Multicolor and OCT image of the study eye at presentation showing the SRF. The SFCT at presentation was 520 µm. (**C**,**D**): Multicolor and OCT image of the study eye at 2-month visit showing the complete resolution of the SRF. The SFCT was 349 µm. (**E**): OCT image of the fellow eye at presentation. The SFCT at presentation was 475 µm. (**F**): OCT image of the fellow eye at the 2-month final follow-up visit. The SFCT was 333 µm.

The SFCT in the study eye showed a significant reduction between baseline and 3rd month follow-up visits in both treatment (*p* < 0.001) and control groups (*p* = 0.001) (Table 2; Figure 1, Figure 2 and Figure 3). 

### 2.4. Effect of Eplerenone on SFCT of the Fellow Eye

The SFCT in the fellow eye showed significant reduction in the treatment group (*p* = 0.005) at the 3rd month visit compared to the baseline. On the contrary, in the control group, no significant reduction in SFCT (*p* = 0.276) was noted between the baseline and 3rd month follow-up visit (Table 2; Figure 1, Figure 2 and Figure 3). During the course of the study, fresh occurrence of SRF was noted in only one (3%) patient in the treatment group and five (17%) patients in the observation group in the fellow eye.

### 2.5. Safety

No serious ocular or systemic side effects related to eplerenone, such as hyperkalemia or hypotension were noted at any of the visits.

## 3. Discussion

The results of this study suggest that oral eplerenone helps in early improvement of visual acuity due to the reduction in the choroidal vascular permeability and faster absorption of the SRF. It reduces the SFCT in the other eye, thereby having some beneficial effects in the fellow eye as well [23]. 

The aim of treatment in acute CSCR is to reduce the choroidal vascular permeability and/or close the RPE leak. Thus, focal laser therapy and photodynamic therapy are considered the mainstay of treatment in acute CSCR. Laser photocoagulation targeted to the extrafoveal leak has been used to close the RPE leak and help in faster absorption of SRF. Focal laser is contraindicated if the leaks are too close to the fovea [24]. Photodynamic therapy (full or reduced fluence) has been found to be effective in reducing the choroidal vascular permeability and SRF. However, it is not widely available or affordable in many developing countries, and its safety effects are still debatable [25,26]. Intravitreal anti-vascular endothelial growth factor agents have been given to reduce vascular permeability in chronic CSCR, although their exact roles in the treatment of acute CSCR are still unclear [27,28,29,30]. Therefore, a specific treatment modality targeting the mechanism of choroidal dysfunction is still required. Oral eplerenone is a systemic mineralocorticoid receptor antagonist which acts on the choroidal mineralocorticoid receptor pathway, thereby reducing the choroidal vascular permeability in CSCR. 

This study demonstrated that oral eplerenone was effective in causing early resolution of SRF. The percentage of patients showing complete resolution of SRF in the treatment group was 45% at the end of one month, while in the observation group, it was 10%. At the end of two and three months, complete SRF resolution achieved in the treatment group was 55% and 62%, respectively. This was higher than that achieved in the observation group (21% at two-month and 31% at three-month follow up). This similar trend is noted in other studies as well. In a study by Zucchiatti et al., 47% of the eyes at the end of one month and 80% of eyes at the end of three months showed complete resolution of SRF in the treatment group [22]. Sun et al. investigated the effects of spironolactone versus observation in patients with acute CSCR [21]. Similar to our study, they found complete SRF resolution was achieved in 56% of the treated cases compared to 8.3% in the observational group at the end of the two-month follow up. Our study results showed statistically significant reduction in SRF height and SFCT in the affected eye in both treatment (*p* < 0.001) and observation (*p* < 0.001) groups through the monthly follow-up visits and also at the 3rd month visit. This shows comparable effectiveness of oral eplerenone in CSCR management to observation. However, oral eplerenone has the additional advantage of hastening the SRF absorption compared to observation. 

In terms of visual acuity improvement, a faster improvement in vision following treatment with oral eplerenone was noted. At the end of two months, all the patients in the study group showed an improvement in visual acuity to 6/6. On the other hand, in the observation group, visual acuity improvement to 6/6 was noted in 100% cases only at the end of three months. 

Significant reduction in the SFCT of the contralateral eye was noted in treatment group (*p* = 0.005) compared to the observational group at the end of three months in our study (*p* = 0.276). 

During the course of the study, only one (3%) patient in the treatment group and five (17%) patients in the observation group showed fresh SRF occurrence in the contralateral eye. This suggests that oral eplerenone may have a beneficial effect in preventing the disease from occurring in the contralateral eye as well. None of the existing studies evaluating the efficacy of oral mineralocorticoid receptor antagonists in acute CSCR have studied its effects on the contralateral eye [21,22]. 

The results of the VICI trial, a multicentric randomized trial comparing the efficacy of oral eplerenone versus observation in eyes with treatment-naïve CSCR, suggested that oral eplerenone was not superior to observation in improvement of visual acuity [20]. It recommended prohibition of the use of oral eplerenone for treating CSCR cases. However, the results from that trial cannot be directly compared with those of this study. The cohort of cases enrolled in this study were cases with first episode of acute CSCR with <3-month duration. Additionally, the trial did not look at the effects of eplerenone on the SFCT of the affected and fellow eyes. 

Our study has the advantage of being a prospective study with patients being followed up at regular monthly intervals for three consecutive months. Our study also has a relatively large sample size compared to other similar studies. The study also looks at the effects of oral eplerenone on the contralateral eye. However, drug safety cannot be established from this study due to the shorter treatment period. One of the main limitations in this study is that the follow-up was relatively short. Considering the short follow-up, we are unable to make any comments about the recurrent attacks in these patients, which are very common. Additionally, we could have done contrast sensitivity and/or micro perimetry assessments, which may have showed a significant change in quality of vision. 

## 4. Materials and Methods

In this prospective case-control study, consecutive patients with a diagnosis of unilateral acute CSCR referred to the retina clinic of Narayana Nethralaya super specialty eye hospital, Bangalore between July 2017 and December 2018 were recruited. All procedures performed were in accordance with the ethical standards of the institutional research committee and with the 1964 Helsinki declaration and its later amendments. Informed consent was obtained from all participants included in the study. Permission for conducting and analyzing the data for this study was obtained from the organization’s institutional review board and ethics committee (C-2019-01-006). 

### 4.1. Inclusion and Exclusion Criteria

Patients were eligible for inclusion if they were ≥18 years of age, with a diagnosis of acute CSCR and were experiencing symptoms for the first time. Acute CSCR was defined by the presence of SRF and visual symptoms for <12 weeks’ duration and with no clinical and/or imaging features of diffuse retinal pigment epithelial changes in the retina. Fluorescein angiography was considered only in cases where the serous detachment raised the possibility of other retinal/choroidal pathologies like age related macular degeneration, polypoidal choroidal vasculopathy, Vogt–Koyanagi Harada disease, posterior scleritis, malignant hypertension, optic nerve pit or choroidal tumors and where the diagnosis of CSCR or presence of associated choroidal neovascularization would not have been possible without angiographic evaluation. Exclusion criteria was the presence of chronic CSCR (duration of visual symptoms more than 12 weeks and/or diffuse retinal pigment epithelial changes) or recurrent CSCR (patients with a history of more than one previous CSCR attack), presence of choroidal neovascularization identified by OCT and confirmed on fluorescein angiography, indocyanine green angiography and/or OCT-angiography; any treatment for retinal disease (including intravitreal injections, photodynamic therapy, laser photocoagulation, vitrectomy), history of other retinal disorders (including age-related macular degeneration, choroidal neovascularization, diabetic retinopathy, uveitis or pathologic myopia). Physician clearance was obtained to identify any contraindications for the use of oral eplerenone. Patients with the presence of any such condition for which eplerenone was contraindicated (such as severe renal, cardiac or hepatic failure, pregnancy, baseline serum potassium > 5.5 mmol/L, concomitant administration of potassium-sparing diuretics, potassium supplements, strong CYP3A4 inhibitors, angiotensin-converting enzyme inhibitors or angiotensin receptor blockers) were not recruited in the study.

All consecutive patients who met the inclusion criteria were randomized alternatively to either the treatment or observation group. The patients in the treatment group were given oral eplerenone 50 mg/day (Table *Eptus* 25 mg) in 2 divided doses for 1 month and was continued until the complete SRF resolution was achieved. Serum electrolytes and creatinine assessments were performed at baseline before starting treatment, at 1 week after commencing the treatment and then at monthly intervals during treatment. Patients were asked to inform us in case they noted any abnormal systemic symptoms following the intake of the drug. Eplerenone was discontinued in the case of abnormal serum electrolytes or creatinine or drug intolerance. 

### 4.2. Examination and Measurements

At baseline, both eyes of each patient underwent assessment of best-corrected visual acuity on Snellen’s visual acuity charts, along with comprehensive ophthalmological examination and multimodal imaging by color fundus photography, fundus autofluorescence and spectral domain-OCT using the Spectralis Heidelberg HRA-OCT machine (Heidelberg Engineering, Heidelberg, Germany). On spectral domain OCT, maximum SRF height was measured manually as the distance between the end of the photoreceptor outer segment layer and RPE. The sub foveal choroidal thickness (SFCT) was defined as the vertical distance between the hyper-reflective line of Bruch’s membrane and the chorio-scleral interface and was measured manually on the enhanced-depth imaging OCT scans. All the measurements were performed during the morning hours from 9 a.m. to 12 p.m. by the same experienced blinded observer (BVH).

### 4.3. Follow-Up Visits

Patients included in the study were followed monthly for 3 months after enrollment in the study. At each visit, both the affected and contralateral eye of each patient underwent best-corrected visual acuity assessment, intraocular pressure measurement, fundus examination and spectral domain OCT examination. The SRF height and SFCT were measured manually at each of these follow-up visits by the single blinded observer (BVH) for both the treatment and control groups. 

### 4.4. Outcome Measures

Percentage of eyes reaching a visual acuity of 6/6 at month 1, 2 and 3 follow-ups in both groups.

- Comparisons between the treatment group and observational group for the SRF height and SFCT in the study eye at every visit for the consecutive 3 months.
- SFCT measurement comparison between the treatment and the observation groups in the fellow eye at each visit.
- Side effects and drug tolerance at each visit.

### 4.5. Statistical TESTS

All statistical tests were performed with GraphPad Prism software (v.8.1.1). The normal distribution of continuous variables was verified with the Kolmogorov–Smirnov test. Results of descriptive analysis for quantitative variables were expressed as mean with standard deviation, while for categorical variables they were expressed as number and percentage. Comparisons between the treatment and observational groups were done using the Fisher exact test and Mann–Whitney U test for categorical and quantitative data, respectively. Comparisons of SRF height and sub foveal choroidal thickness between the three time points were performed using repeated measures analysis of variance (ANOVA) with Geisser–Greenhouse correction for post hoc analysis. Comparison between the baseline visit and each of the monthly visits for SRF height and sub foveal choroidal thickness was done using the Wilcoxon matched-pair rank sum test. In all analyses, p values < 0.05 were considered statistically significant.

### 4.6. Declarations

**Ethics approval and consent to participate:** All procedures performed in studies involving human participants were in accordance with the ethical standards of the institutional research committee (Narayana Nethralaya institutional review board, C-2019-01-006) and with the 1964 Helsinki declaration and its later amendments or comparable ethical standards.

**Consent for publication:** Informed consent was obtained from all individual participants included in the study. The authors certify that they have obtained all appropriate patient consent forms. In the form, the patient has given his consent for his/her images and other clinical information to be reported in the journal. The patients understand that their names and initials will not be published and due efforts will be made to conceal their identity, but anonymity cannot be guaranteed. 

## 5. Conclusions

Our study shows that oral eplerenone is an effective, non-invasive treatment option having a favorable short-term safety profile in the treatment of acute CSCR by achieving faster anatomical and functional improvements. Further studies evaluating oral eplerenone in acute CSCR with long-term follow-up can be considered. 

## Figures and Tables

**Table 1:** Characteristics between the treatment group and observation group in acute CSCR.

Variable	Treatment Group (N = 29)	Observation Group (N = 29)	*p* Value
Age (years)	40.4 ± 7.1	43.3 ± 8.34	0.24
Sex (M:F)	23:6	25:4	0.73
Laterality (RE:LE)	18:11	17:12	>0.999
Mean duration of symptoms (weeks)	6.46 ± 1.45	5.87 ± 2.09	0.897
Visual acuity at presentation (N, %)	6/6–6/12—17,57 6/18–6/36—11,38 ≤6/60—1,3	6/6–6/12—20,69 6/18–6/36—9,31 ≤6/60—0,0	
SRF height (µm) at baseline	307 ± 234	381 ± 227	0.146
SFCT study eye (µm) at baseline	479 ± 58.6	443 ± 97.8	0.144
SFCT contralateral eye (µm) at baseline	406 ± 106	340 ± 92.3	0.060
Eyes with complete SRF resolution at month 1	13/29 (45%)	3/29 (10%)	0.007
Eyes with complete SRF resolution at month 2	16/29 (55%)	6/29 (21%)	0.014
Eyes with complete SRF resolution at month 3	18/29 (62%)	9/29 (31%)	0.034
Improvement in visual acuity to 6/6 at month 1	27/29 (92%)	21/29 (74%)	0.079
Improvement in visual acuity to 6/6 at month 2	29/29 (100%)	25/29 (86%)	0.112
Improvement in visual acuity to 6/6 at month 3	29/29 (100%)	29/29 (100%)	>0.999

Abbreviations: M—male; F—female; SRF—subretinal fluid; SFCT—sub foveal choroidal thickness.

**Table 2:** Changes in the SRF height and SFCT from baseline through month three for both treatment and observation groups.

Variable	Group	Baseline	Month 1	*p* Value (Wilcoxon)	Month 2	*p* Value (Wilcoxon)	Month 3	*p* Value (Wilcoxon)	*p* Value ANOVA
SRF (µm)	Treatment	307 ± 234	87.9 ± 101	<0.000	64.4 ± 87.6	<0.000	61.7 ± 84.8	<0.000	<0.000
	Observation	381 ± 227	217 ± 128	<0.000	147 ± 121	<0.000	116 ± 115	<0.000	<0.000
SFCT study eye (µm)	Treatment	479 ± 58.6	395 ± 75.6	<0.000	387 ± 82.6	<0.000	385 ± 97.7	<0.000	<0.000
	Observation	443 ± 97.8	407 ± 78.3	0.032	390 ± 78.5	0.003	378 ± 74	0.000	0.001
SFCT contralateral eye (µm)	Treatment	406 ± 106	372 ± 80.7	0.025	354 ± 83.4	0.004	340 ± 90.9	0.004	0.005
	Observation	340 ± 92.3	321 ± 80.4	0.007	336 ± 85.3	0.402	343 ± 87.8	0.987	0.276

Abbreviations: SRF—subretinal fluid; SFCT—sub foveal choroidal thickness.

## Abbreviations

CSCR	central serous chorioretinopathy
SRF	subretinal fluid
OCT	optical coherence tomography
RPE	retinal pigment epithelium
SFCT	subfoveal choroidal thickness
